# Protein Adaptations in Archaeal Extremophiles

**DOI:** 10.1155/2013/373275

**Published:** 2013-09-16

**Authors:** Christopher J. Reed, Hunter Lewis, Eric Trejo, Vern Winston, Caryn Evilia

**Affiliations:** ^1^Department of Chemistry, Idaho State University, Pocatello, ID 83209, USA; ^2^Department of Biological Sciences, Idaho State University, Pocatello, ID 83209, USA

## Abstract

Extremophiles, especially those in Archaea, have a myriad of adaptations that keep their cellular proteins stable and active under the extreme conditions in which they live. Rather than having one basic set of adaptations that works for all environments, Archaea have evolved separate protein features that are customized for each environment. We categorized the Archaea into three general groups to describe what is known about their protein adaptations: thermophilic, psychrophilic, and halophilic. Thermophilic proteins tend to have a prominent hydrophobic core and increased electrostatic interactions to maintain activity at high temperatures. Psychrophilic proteins have a reduced hydrophobic core and a less charged protein surface to maintain flexibility and activity under cold temperatures. Halophilic proteins are characterized by increased negative surface charge due to increased acidic amino acid content and peptide insertions, which compensates for the extreme ionic conditions. While acidophiles, alkaliphiles, and piezophiles are their own class of Archaea, their protein adaptations toward pH and pressure are less discernible. By understanding the protein adaptations used by archaeal extremophiles, we hope to be able to engineer and utilize proteins for industrial, environmental, and biotechnological applications where function in extreme conditions is required for activity.

## 1. Introduction

 Archaea thrive in many different extremes: heat, cold, acid, base, salinity, pressure, and radiation. These different environmental conditions over time have allowed Archaea to evolve with their extreme environments so that they are adapted to them and, in fact, have a hard time acclimating to less extreme conditions. This is reflected in current taxonomy in Archaea [1, 2]. Archaea are presently partitioned into four branches: the halophiles, the psychrophiles, the thermophiles, and the acidophiles. While we typically think about the methanogens as a distinct group, they are, in fact, spread among all the other branches in Archaea. For the purposes of this review, we have included them in their principle branch (e.g., the thermophiles) where appropriate.

The branches of Archaea intersect in interesting ways. For example, alkaliphiles (which are not one of the branches mentioned above) are grouped with the halophiles because the two archaeal groups not only are found together in saline environments but also share genome similarities. Thermophiles and acidophiles branches are also clustered together, not only because most acid environments are hot but because these groups also share genome similarities. Many archaeal piezophiles (pressure-loving organisms) are found at deep sea thermal vents, leading them to have many similarities to hyperthermophiles. Psychrophiles also share branches with the halophiles; again for similar reasons, psychrophilic environments can be hypersaline. 

 In researching different Archaea, we have divided Archaeal protein adaptations into three general categories: thermophilic, psychrophilic, and halophilic. Organisms that could be classified into more than one branch could show one or more of these three major adaptations with minor adjustments to accommodate environmental conditions. For example, haloalkaliphiles, like *Natronomonas pharaonis*, have their primary protein adaptation as halophilic with no clear adaptation for the extreme basic environment (pH > 11) in which they live [3]. For *N. pharaonis*, this fits with their cell conditions, which are only mildly basic but extremely saline. *N. pharaonis* allows salt across the cell membrane, for a high internal salt concentration, but the intracellular pH is mildly basic (pH ~ 8).

Acidophilic proteins, some of which show increased negative surface charge, also show thermophilic adaptations [4, 5]. Acidophiles pump protons out of the cell to maintain a mildly acidic cytoplasm. pH varies from 5.0 to 6.5, which allows their primary adaptation to be for their thermophilic environment. Mesophilic acidophiles prove this point as their proteins have small changes that could account for their activity in the acidic cytoplasm [4, 5]. 

Thermophiles, psychrophiles, and halophiles, on the other hand, have evolved to live within their environmental conditions, rather than to adapt ways to circumvent it. Obviously, thermophiles and psychrophiles cannot shut out heat or cold, so, besides cellular adaptations like secondary metabolites which maintain overall cell stability, this required novel protein adaptations to survive. Halophiles had to evolve a system to deal with extreme osmotic stress. To facilitate this, they possess a membrane system to pump potassium in while pumping sodium out [6]. The intracellular concentration of potassium, depending on which species of Halobacteria, can vary from 1.2 to 4.5 M [6–9]. This functions to maintain the osmotic balance in the cell. However, their proteins require certain features that allowed them to work under such extreme ionic conditions. 

While the three categories (thermophiles, psychrophiles and halophiles) of Archaea show the most obvious protein adaptations to their environments, those adaptations are not necessarily uniform throughout all of their proteins. This makes studying protein adaptations in all extremophiles, especially Archaea, difficult because one is not simply looking for a single trend or feature. In fact, variability in adaptations has been noted multiple times throughout studies of archaeal and extremophile proteins [4, 12–14]. There have been many reasons proposed for these differences. One of the more convincing ideas is that, by only having a few protein modifications, the enzyme might have activity over a range of conditions [4, 14–17]. This gives the organism some flexibility to grow in a range of different conditions. Another idea is that having various protein adaptations could be an alternative to regulatory pathways. Along these lines, a particular protein would become optimally active only under certain conditions, which would save the organism from having to regulate that protein through cell signaling pathways [18]. This supports the notion that Archaea take advantage of simple adaptations to reap the benefits of their extreme environments. 

Not all adaptations are hard coded into the protein sequence though. This follows because, in order for proteins to function under extreme conditions, multiple structural considerations must be accommodated to balance activity, flexibility, and stability [19–21]. Some protein structure/function issues under environmental extremes can be accommodated by flexible folding [22]. Protein folding states are dynamic; they have to change in order for the protein fold to accommodate different conditions and remain active. However, there is a limit to how many folding events can be accomplished in order to meet environmental challenges [23, 24]. For example, the cysteinyl-tRNA synthetase from *Halobacterium salinarum* sp. NRC-1 shows little change in activity and global structure when the salt concentration varies from 3.5 M to 2 M. This change would be a large decrease in salinity for the organism, which would cause it to lose the integrity of the cell membrane and S-layer, but this enzyme is tolerant of the change. The enzyme probably remains active and structurally sound due to local folding events that accommodate the change in conditions. When the salinity is further decreased from 2 M to 0.5 M, the enzyme loses activity and its structure, indicating that folding states have their limit and other forces need to be at work to get the enzyme to remain stable [16, 18]. This reflects that sequence changes over time have led to protein features that protect or preserve a function under greater extremes. 

In this review, we will summarize the current known protein adaptations for thermophilic, psychrophilic, and halophilic Archaea. Along the way, we will discuss other extreme conditions, such as acid, base, and pressure, for which their adaptations are considered secondary to that of the main adaptation. For example, thermoacidophiles, thermopiezophiles, and haloalkaliphiles will be discussed with thermophiles and halophiles. This was done as an attempt to sort out “minor” adaptations into their defining category while not ignoring them. 

## 2. Thermophilic Proteins

While thermal vents and hot springs are considered to be some of the most extreme environments on Earth, several organisms are able to thrive in these hostile locations where most life would perish. Among these are thermophiles and hyperthermophiles. While the two share similar adaptations to survive in these extremes, they differ in their temperature growth optimum. Hyperthermophiles can grow optimally up to 105°C, whereas thermophiles are classified as growing between 50°C and 70°C. At such extreme temperatures, proteins lacking the necessary adaptations undergo irreversible unfolding, exposing the hydrophobic cores, which causes aggregation [25]. Thermophilic proteins have several adaptations that give the protein the ability to retain structure and function in extremes of temperature. Some of the most prominent are increased number of large hydrophobic residues, disulfide bonds, and ionic interactions.

### 2.1. Oligomerization and Large Hydrophobic Core

Observed within many thermostable proteins are deviations from standard quaternary organization seen in their mesophilic counterparts. This strategy is thought to increase the rigidity of the individual subunits, promote tighter packing of the hydrophobic core, and reduce exposure of hydrophobic residues to solvent [14]. Three acetyl-CoA synthetases and one amylase from thermophilic Archaea highlight the argument that aberrancies in quaternary structure are the causative agents in these enzymes' thermostability and others as well.

Recent characterizations of two acetyl-CoA synthetases (ACS) from *Ignicoccus hospitalis* [26] and *Pyrobaculum aerophilum* [27] have uncovered novel structural adaptations, namely, a difference in oligomeric state from that of the mesophilic variants. Compared to their mesophilic counterparts, these hyperthermophilic enzymes form octomers whereas the aforementioned mesophiles follow the general trend of being monomers or homodimers. However, the ACS from *Archaeoglobus fulgidus*, a hyperthermophile with lower temperature growth optimum, is a trimer [28].

The hydrophobic effect becomes increasingly more important in protein stability and folding as temperature increases. This has been observed in a phosphotriesterase from *Sulfolobus solfataricus* where tighter packing is observed due to favorable hydrophobic interactions at the dimer interface [29]. This strategy could have also been adopted by hyperthermophilic ACS proteins in order to decrease the overall ratio of surface area to volume in regard to individual subunits and solvent exposed hydrophobic regions. As a consequence this would result in tighter packing of the hydrophobic core, a general feature of all thermostable proteins. 

Contrary to the increase in oligomeric state in ACS, the study of a thermostable amylase from *Pyrococcus furiosus* (Pf) showed a lack of oligomerization compared to the mesophilic homologues [30]. This is the first example of a functional monomeric version of a cyclodextrin hydrolyzing enzyme. Bacterial homologues require dimerization before activity is seen [31]. A novel domain on the N-terminus (N′) allows Pf amylase to be active as a monomer, although as a consequence it lacks the transferring activity seen in CD-hydrolyzing enzymes. The bacterial N-domain possesses a loop that extends over the active site that acts as structural “lid” and functions to stabilize certain substrates such as maltose, whereas Pf amylase does not. 

While the absence of quaternary structure and the N-terminal loop in Pf amylase alters substrate specificity, it would appear that this modification is important in regard to overall stability. It is widely accepted that structural flexibility in protein structure is unfavorable in thermostable enzymes, even though there is no generally accepted mechanism by which rigidity and by proxy stability is achieved [32]. In the case of thermostable ACS, a higher oligomeric state is favorable, whereas Pf amylase implements the opposite strategy: organizing all the necessary components into a single subunit, creating structural rigidity, and promoting tighter packing of the hydrophobic core. Both cases lend credence to the hypothesis that changes in quaternary structure can be advantageous; however, there is no discernible trend within this strategy.

### 2.2. Increased Number of Disulfide Bonds

Disulfide bridging between cysteine residues is an important tertiary structural element that is paramount in determining the overall structure of a protein. Organisms within all domains of life have adapted their own systems of keeping proper bridging in check, as some are favorable and some completely inactivate enzymes. Within thermostable enzymes these structural elements are significant, since they have been shown to increase stability within thermophilic proteins and play a role in preventing alteration of quaternary structure. Studies by Cacciapuoti et al. [12, 33] and separately by Boutz et al. [34] provide evidence for these claims.

One example of the use of disulfide bridging in thermostability is 5′-deoxy-5′methylthioadenosine phosphorylase II which was used to study the CXC motif and intrasubunit disulfide bonds within thermophilic proteins [12, 33]. Using circular dichroism spectroscopy, under reducing conditions, the hexameric protein was seen to disassociate into its monomeric state in a reversible fashion. Chemical and thermal denaturation resulted in irreversible degradation of structure. Single and double mutants within key cysteine residues demonstrated an appreciable change in thermostability. The native protein was observed to be almost completely denatured at 108°C, whereas the single mutant (C262S) was shifted to 102°C and 99°C for the CXC double mutant (C259S/C261S). These results elucidated that mutations within these residues decreased thermal stability inferring that disulfide bridging is a structural adaptation [12]. It is noteworthy that the CXC motif forms a strained 11-member disulfide ring, which has been implicated as a useful redox reagent [35]. This novel adaptation parallels that of disulfide isomerases, whose function is to maintain proper disulfide bridging within proteins [36]. 

Disulfide bonds have also been shown to be important in oligomerization. The citrate synthetase from *Pyrobaculum aerophilum* illustrated the use of disulfide bonds to create cyclized protein chains that topologically interlinks two monomeric subunits of the homodimer [34]. This novel structural feature confers stability within the dimer by disallowing separation of the individual subunits. These two examples demonstrate the role of disulfide bridging in thermostability, either from increased rigidity or the interlocking of adjacent chains between monomeric subunits. 

### 2.3. Increased Salt-Bridging

Salt-bridging is a prevalent feature of most thermophilic enzymes compared to their mesophilic variants [37]. This is in contrast to findings that salt-bridging may destabilize mesophilic proteins and are unfavorable compared to hydrophobic interactions [38]. The desolvation penalty and entropic cost associated with ion pairing found in salt bridges is more easily overcome at higher temperatures [39]. When these thermodynamic considerations are negated, salt bridges become a structurally stabilizing element, increasing the thermal capacity of proteins using favorable charge-charge interactions.

Experimental circular dichroism studies within a thermophilic ribosomal protein, L30e, from *Thermococcus celer* produced a noticeable change in thermal capacity without causing major structural changes [39]. Mutations from charged residues involved in salt-bridging to hydrophobic residues increased the heat capacity change of unfolding, Δ*C*_*p*_. Lowering of Δ*C*_*p*_ could be a strategy used to increase thermostability in proteins, favoring the natively folded state over that of the unfolded. This demonstrates that favorable interactions of charged residues (salt bridges) improve the thermal stability of proteins [39].

### 2.4. Increased Surface Charges

Ubiquitous within thermostable proteins is the increase of charged residues on the surface of proteins [40]. Replacement of polar uncharged surface residues with polar charged residues can result in an overall increased stability due to several factors. At higher temperatures, polar residues such as asparagine and glutamine can undergo deamination which would reduce stability [40]. Replacement of these and other thermolabile residues increase both short- and long-range charge interactions which, generally, help to protect against thermal denaturation [41].

To further explain the roles of charge-charge interactions outside of those involved in salt bridges, multiple single-point mutations of surface charged residues to alanine were made on the ribosomal protein, L30e, from *Thermococcus celer* [41]. It was found that the thermal capacity of this thermophilic protein could be further increased by favorable mutations to charged residues and could be decreased when surface charges were replaced with alanine. Long-range charge-charge interactions were found to be a large determining factor in the stability of L30e, as removal of these electrostatic interacts caused greater susceptibility to thermal and chemical denaturation. 

The effects of too much surface charge were observed in a putative DNA binding protein from *Methanothermobacter thermautotrophicus*, MTH10b [42]. While the protein has an unknown activity, the thermal capacity was greatly reduced in the absence of salt. In the crystal structure a highly charged surface was observed, which provided insight to the salt-dependent stability. Uneven surface charge distribution, the vast majority of which is positively charged, is attributed with causing the protein to lack the intrinsic thermostability possessed by others within this protein family. MTH10b demonstrates that the presence of salt acts to offset the repulsive forces that act to destabilize the protein bestowing thermostability [42]. 

While higher content of surface charged residues can serve to stabilize proteins by preventing aggregation at higher temperatures, it can also serve to destabilize the structure [43]. The necessity of inorganic salts for protein stability and functionality has also been observed for other enzymes in *M. kandleri* [44]. This suggests that an extremely charged protein surface may require some form of compensation in order to reap the positive benefits of this structural adaptation.

### 2.5. Industrial Applications

Thermophilic enzymes show a high potential for biotechnological and industrial application because they are optimally active at high temperatures, where the kinetics and thermodynamics of the catalyzed reaction are more favorable [45]. This allows for a more efficient reaction and higher product yield. Secondary benefits that accompany thermostability include a lower chance of bacterial contamination (important in food and drug applications) and the reduction of operating costs from constant enzyme replacement due to thermal denaturation [45, 46]. The first application of thermophilic enzymes was Taq DNA polymerase from the bacterium *Thermus aquaticus*. The clever use of this enzyme reduced the cost and allowed the automation of PCR, which greatly advanced research in biochemistry and molecular biology laboratories [45, 46]. Today, a number of thermophilic DNA polymerases from archaeal species are used instead of *T. aquaticus* including *pfu*Turbo, DeepVent_R_, Therminator, among others (Stratagene Inc., and New England BioLabs Inc.). A potential application of archaeal thermophilic enzymes was discovered in mutational studies done on a thermostable amylase from *Pyrococcus furiosus*. A mutation in Pf amylase caused an increase in the production of maltoheptaose from *β*-cyclodextrin. Maltoheptaose and other linear maltooligosaccharides are of high value in the food, cosmetic, and pharmaceutical industries where they can be used as carriers [30]. Other potential uses of thermophilic enzymes exploit not their activity at high temperatures but their lack of catalytic activity at ambient temperatures. Thermophilic enzymes can act as optical nanosensors which could bind a substrate but not turn over a product [46]. The substrate-enzyme complex could then be detected by measuring a variation of enzyme fluorescence, which in turn could allow for quantification of the amount of substrate in a sample. Such innovations have the potential to become important tools in biotechnology, medical testing, and drug discovery [46]. 

## 3. Piezophilic Proteins

Piezophiles are organisms that live under extremely high hydrostatic pressure often in other extremes, like high or low temperature. Their typical habitat is deep in the ocean, under extreme pressure, and in the extreme heat of hydrothermal vents or in the cold of the ocean. Most archaeal piezophiles, such as *Pyrococcus abyssi* or *Sulfolobus solfataricus*, are thermophilic, while psychrophilic piezophiles are usually, but not strictly, bacterial [47]. Adaptations in their proteins to the extreme pressure appear to be secondary to their adaptations to temperature [48]. The general adaptations for archaeal and bacterial piezophiles, outside of their temperature adaptation, are a compact, dense hydrophobic core, the prevalence of smaller hydrogen-bonding amino acids and increased multimerization [49–51].

One example of this is seen in* Pyrococcus abyssi*, a hyperthermophilic piezophile. There is an increase in small amino acids across its proteome when compared to that of the related archaeon but non-piezophile, *Pyrococcus furiosus* [50]. Overall reduced amino acid size leads to a reduction in the number of large hydrophobic residues, such as tryptophan and tyrosine, in the core of its proteins. This is contrary to the composition of the hydrophobic core seen in most thermophilic proteins, which contain a higher percentage of large amino acids. Nevertheless, such an adaptation is advantageous because it allows for tighter packing, creating a more pressure stable protein [50]. Another example of piezophilic adaptation of a compact hydrophobic core was a study done with the Sso7d, a DNA binding protein from *Sulfolobus solfataricus* (Ss) [52, 53]. Using mutagenesis and structural studies with NMR, it was demonstrated that any change, that either created a cavity in the protein or disrupted the hydrophobic nature of the protein's core, decreased the pressure stability as well as the thermostability of the protein [52, 53]. Similar results were seen in glutamate dehydrogenase from *Thermococcus litoralis* [54]. 

Another way proteins can cope with pressure is to form multimeric proteins. The piezophlic protein, TET3 peptidase (TET3) from *Pyrococcus horikoshii*, forms a discreet dodecamer, rather than a barrel-shaped multimer, and demonstrates increased stability at high pressure [55]. The fact that it formed a dodecamer was important for this protein, since its formation makes the individual monomers more compact in shape. By making its monomers more compact, there is less chance for water to penetrate the core of the protein when high pressures are applied. The trapped water would then disrupt the structure of the protein [49, 55]. 

Multimerization also protects the hydrogen bonding between the protein subunits, which, in general, are not as susceptible to pressure as ionic interactions [49, 55]. Ionic interactions, especially electrostatic interactions, are more susceptible to solvation which disrupts these intraprotein interactions at higher pressure [49, 51]. The strength of the hydrogen bonds between the subunits is enough to mitigate the salt bridge instability [55]. Some thermophilic adaptations, such as an increase in basic amino acids, especially arginine, were found to be beneficial for a protein in both extreme environments. This has also been observed in proteins from *P. abyssi* [50]. 

While archaeal psychrophilic piezophiles do exist, they are relatively unstudied in terms of their protein adaptations. However, the bacteria that do occupy this niche have important and sometimes similar adaptations to thermopiezophiles. In particular, psychrophilic piezophiles do not rely on salt bridges for protein stability, like the thermopiezophiles, which helps them adapt to low temperature and high pressure [48].

### 3.1. Possible Industrial Applications

Little research has been done on piezophilic enzymes; however, there is great potential industrial applications. There are many industrial processes that use high pressure coupled with high or low temperature, especially within the food industry. High pressure is not only sterilizing but also preserves the color and flavor of foods. Enzymes isolated from psychrophilic or thermophilic piezophiles could function under these conditions [56].

Another potential to exploit with piezophilic proteins is the bias in chemistry that has been seen with some piezophilic enzymes. Abe and Horikoshi discussed a porcine *α*-amylase that demonstrated a higher production of maltotriose instead of maltose and maltotetraose, when maltohexaose was used as a substrate in high pressure [57]. Conversely, in low pressure, all three products were produced at the same rate [57]. Other piezophilic enzymes could show similar properties, and this could be exploited to select for certain products that are applicable to industry.

## 4. Acidophilic Proteins

Acidophiles are defined as organisms that grow in the lower extremes of pH. Acidophilic enzymes have optimal structure and stability in acidic environments and have been shown to be catalytically active at pHs as low as 1. Most known acidophiles are also thermophiles, and hence their proteins reflect thermophilic features. Interestingly, the adaptation of acidophilic proteins to pH is unclear and inconsistent. 

Acidophilic proteins must adapt to the low pH because acid interferes with the charge on many residues. At low pH many polar charged residues become protonated and, therefore, their charges change. This has the possibility of disrupting stabilizing structural interactions, unfolding the protein.

While the specific adaptation has not been explored in great detail, the activity of these proteins at low pH seems to be attributed to the prevalence of acidic (negatively charged at a neutral pH) amino acids on the surface of these enzymes and proteins. 

### 4.1. Negative Surface Charge

Research has shown that a number of acidophilic enzymes have optimal activity at a pH significantly lower than the intracellular pH where that enzyme is located. One explanation for pH stability is offered from research conducted on acidophilic and thermostable endo-*β*-glucanase from *Sulfolobus solfataricus*. This enzyme has an optimum pH of 1.8 [58]. A prominent feature is the excess of glutamic and aspartic acid surface residues on the enzyme when modeled. This results in a highly negative surface at a pH of 7. Huang et al. also noted that many acidic surface residues have been attributed to instability at high pH because of the repulsion of these excess negative charges. However, at a lower pH of 2, endo-*β*-glucanase would not have the excess negative charge seen at higher pH, which could help stabilize it in the acidic conditions. These extra acidic residues would also correspond to a lower isoelectric point (pI) for the endo-*β*-glucanase. However, it should be noted that nonacidophilic *β*-glucanases have theoretical pI values very similar to that from *S. solfataricus*, while having optimal activity at neutral to only slightly acidic pH. This suggests the abundance of acidic surface residues cannot be the only factor in determining acid stability of endo-*β*-glucanase [58].

### 4.2. Possible Explanations for Discrepancies in pH Optima

Another example of low pH stability was demonstrated by the *α*-glucosidase from *Ferroplasma acidiphilum*. *α*-Glucosidase demonstrated a preference for pH of 3 instead of 5.6, which is the internal average cytoplasmic pH of *F. acidiphilum* [59]. Similarly carboxylesterase in *F. acidiphilum* was also shown to have a pH optimum of approximately 2. Several other cytoplasmic enzymes also showed similar pH optima. All of these enzymes showed significantly lower activity after the pH was higher than 5, except *F. acidiphilumα*-glucosidase which was still ~60% active [59]. Such low pH optima would be expected in excreted enzymes, due to the acidic environment they are subjected to, but not enzymes that are in the cytoplasm or membrane. Golyshina and Timmis proposed two possible explanations for these discrepancies. It is possible that these enzymes are localized to highly acidic “compartments” within the cytoplasm, even though there is little evidence to support this claim. Another suggestion is that these enzymes form multienzyme complexes which raise the pH optima closer to that of the cytoplasm (5.8). However, no multienzyme complexes like those have been observed in *F. acidiphilum* or other acidophiles [59].

Not all proteins from acidophiles have a preference for low pH as seen in the previous examples. This would be expected since the intracellular pH is not as acidic as external environment. An example of this is seen in the ATP-dependent DNA ligase in *Ferroplasma acidarmanus*. While the glucosidase and other enzymes from *F. acidiphilum* have a pH optima ranging from 2 to 3, *F. acidarmanus* DNA ligase prefers a more neutral environment. It has optimal nick joining activity at pH 6-7, which is similar to DNA ligases from nonacidophiles [60, 61]. This begs the question why some intracellular acidophilic enzymes have such a low pH optima while others, like the DNA ligase, do not. The answer could be related to the substrate of the enzyme; DNA has decreased stability at acidic pH [60, 61]. Therefore, it would be disadvantageous for the *F. acidarmanus* DNA ligase to be optimally active at a low pH.

### 4.3. Possible Industrial Applications

Many of these acidophilic enzymes also fall into the thermophilic category and have potential for biotechnological and industrial applications. One such example is in biofuels production where currently high sugar compounds (e.g., corn) are used for ethanol production. Polymeric and oligomeric sources provide a large but unfortunately inaccessible carbon source. For example, if cellulases and xylanolytic enzymes could be used in a hot acidic environment, then the high temperature and acidity could help hydrolyze lignocellulosic materials, making them more accessible [5]. This could help improve ethanol yields from these carbon sources. Another potential application could be in the food industry where glucoamylases could be used to break down complex polysaccharides into basic dextrose and fructose sugars [5]. If these enzymes were heat and acid stable, this could improve the efficiency of monosaccharide production. Further applications of thermal/acid stable enzymes could be in mining industries. The release of acid and metal contaminants from mining sites could damage the environment [4]. The technique known as bioleaching utilizes microorganisms and their enzymes to harvest metals such as copper, nickel, cobalt, zinc, and uranium [62]. This could reduce the environmental damage done by these mining operations.

## 5. Psychrophilic Proteins

Psychrophiles are a class of extremophiles that grow at temperatures below 20°C [63]. A majority of research on protein adaptations in psychrophiles has been done with bacterial and eukaryotic proteins [64]. Nevertheless, there have been a number of studies that have been done on archaeal organisms living in extremely cold environments; most of the research on archaeal psychrophiles has been done on methanogens growing in Alaska and the Antarctic [65].

A typical protein has extremely low activity at temperatures below 20°C, which is unsuitable for a growing cell [66]. Enzyme activity decreases at lower temperatures due to a lower mean kinetic energy; the conformational movements of a protein become slower and therefore enzymatically less efficient [67]. Also, at low temperatures, the energy barrier of activation for catalysis becomes too great for a protein, further reducing the enzyme's activity [66]. Adaptations in psychrophilic proteins allow them to have enough activity in low temperatures for psychrophilic organisms to thrive in the cold, even though the optimal activity for these proteins is at a temperature above their physiological temperature [66]. Psychrophilic proteins have high activity at low temperatures because they are better able to move and change conformation due to a structure that is more flexible [64]. 

### 5.1. Weak Protein Interactions

Psychrophilic proteins have greater flexibility due to a lower energy barrier between the various conformations of the protein [66]. This is because of a difference in the amino acid composition from mesophilic proteins. In general, the stabilizing interactions typically found within a protein are weakened or removed in cold-active proteins. In an excellent review of cold- and heat-active enzymes by Feller, the following adaptations in psychrophilic proteins were summarized: (i) glycine residues are increased, which provide greater conformational mobility in psychrophilic proteins, (ii) proline residues, which provide conformational rigidity, are reduced in loop regions, (iii) salt bridge and hydrogen bond forming arginine residues are reduced, (iv) the size of nonpolar residues in the protein core is reduced to create weaker hydrophobic interactions [66]. As an example of these features, proteins from the archaeal cold-adapted halophile *Halorubum lacusprofundi* display a decrease in large hydrophobic amino acids, such as tryptophan, and in hydrogen bond forming residues, like glutamic acid. In the *H. lacusprofundiβ*-galactosidase, there was an increasing hydrophobicity observed on the protein surface, which replaced anionic electrostatic interactions which are usually abundant on halophilic proteins [68, 69]. These types of amino acid trends have also been reported in the elongation factor 2 proteins of psychrophilic methanogens [70].

Genomes from a number of archaeal methanogens across a wide range of optimal growth temperatures were examined by Saunders and coworkers in 2003. Using the draft genome sequences from two psychrophilic methanogens, *Methanogenium frigidum* and *Methanococcoides burtonii*, three-dimensional models of proteins were constructed and compared to other modeled proteins from mesophilic and thermophilic methanogens [71]. As expected, a decrease in the number of charged residues on the amino acid surface was observed on the cold-adapted proteins. Furthermore, an increase in glutamine and threonine residues was seen in these proteins. This is thought to reduce the charge on the protein surface without causing aggregation by creating a surface that is too hydrophobic [71]. This research was examining psychrophilic adaptations in a large number of molecular models (141), and it supported adaptations that have been seen in studies from single proteins [71]. 

### 5.2. Lower Thermal Stability

Weaker interactions between amino acid residues in a psychrophilic protein prevent it from being “frozen” in a particular conformation and make the molecular motions needed for catalysis possible. A consequence of these weaker interactions is a less stable protein; thus, cold-adapted proteins unfold at lower temperatures than mesophilic proteins [66, 72, 73]. The thermal unfolding of psychrophilic proteins has been reported to occur through a single transition. This is because the weaker interactions in cold-adapted proteins have greater influence on the overall stability, and local unfolding greatly destabilizes the protein due to fewer stabilizing interactions [66]. These characteristics have been observed in an archaeal cold-shock protein from *Methanogenium frigidum*, which was shown to be less stable at its optimal temperature than its mesophilic homologue from *E. coli* [74].

### 5.3. Increased Specific Activity

The catalytic activity of a psychrophilic enzyme, due to the more flexible structure, is much greater at low temperatures than the same enzyme from a mesophile. In fact, despite decreased reaction rates at low temperatures, the specific activity (*k*_cat_) of a psychrophilic enzyme is typically 10 times greater than a mesophilic enzyme [66, 73]. A typical observation that is made to explain the greater *k*_cat_ is the increase in binding site size in psychrophilic proteins [64]. In psychrophilic enzymes, the substrate binding area is enlarged by a number of mechanisms while the catalytic residues are unchanged [66]. Some of the mechanisms by which this area is enlarged include deletion of loops near the binding site [75], strategic glycine residues near the functional sites [66], and pulling the protein backbone out to increase substrate accessibility [76]. As a result, substrates are not able to bind as well to a psychrophilic enzyme, and, therefore, the Michaelis-Menten constant (*K*_*m*_) of psychrophilic enzymes is high [66, 77]. Poor substrate affinity improves enzyme activity at low temperatures because it reduces the energy of activation for the enzyme [66]. 

### 5.4. Industrial Applications

Psychrophilic enzymes have found useful applications in the biotechnical industry. Due to their higher activity at low temperatures, cold-adapted lipases from bacterial psychrophiles are used in commercial detergents [63]. Likewise, cellulases find use due to their reduced thermal stability, making it easier to inactivate the enzyme after a certain amount of time. This is important for stone washing in the textile industry, where if the cellulases are active too long, the mechanical resistance of the cotton is lost [63, 78]. Archaeal cold-adapted enzymes are not as widely used as enzymes from bacteria. Nevertheless, they still have many possible applications in industry due to their adaptations.

## 6. Halophilic Proteins

Salt has significant effects on the solubility, stability, and conformation of a protein, which ultimately affects its ability to function. Organisms that thrive in extremely salty environments like the Great Salt Lake or the Dead Sea have two major ways through which they adapt to the extreme salt. Some halophiles, mostly halophilic bacteria and eukaryotes, prevent the entry of the inorganic salts (such as NaCl) into the cell and synthesize small organic molecules (like ectoine), known as osmolytes, to balance the osmotic pressure [8]. Halophilic Archaea, though, survive by taking in high concentrations of inorganic salts, requiring their proteins to carry adaptations that allow them to remain stable and functional. At high salt concentrations (higher than 0.1 M), water is less available to protein because most water is surrounding salt in an ionic lattice [8]. The lower availability of water can cause hydrophobic amino acids in a protein to lose hydration and aggregate. Therefore, high salt concentrations strengthen hydrophobic interactions in a protein. Salt also interferes with the electrostatic interactions between charged amino acids [79]. Nonhalophilic proteins cannot function in high salt concentrations because the hydrophobic and electrostatic interactions they normally rely on for proper folding and for maintaining stability are greatly altered. This can even lead to destabilization of the protein, potentially causing global unfolding and aggregation, ultimately leading to precipitation. Archaeal halophilic proteins have a number of adaptations that allow them to utilize the high concentrations of inorganic salt to stabilize their native fold.

### 6.1. Increased Acidic Residues

One of the most notable differences between halophilic and nonhalophilic proteins is the large increase in acidic residues, like glutamic and aspartic acid, on the protein's surface. This is almost ubiquitous with halophilic proteins and can distinguish between halophilic and nonhalophilic protein sequences [80]. There are a number of possible roles for these acidic residues. It is thought that the increased negative charge on the protein's surface allows the protein to compete with ions for water molecules and, therefore, keep the protein in solution [79, 81–83]. This is supported by the crystal structures of halophilic proteins that show water binding with these acidic surface residues [8, 83, 84]. Bioinformatics analysis of halophilic proteins has shown that their sequences also consistently contain less serine. Serine is good at interacting with water but not at competing with charged ions, so it is thought that serine is less useful for proteins at high salt concentrations [85]. An alternative to increased water binding would be that the acidic residues on halophilic proteins bind hydrated cations which would maintain a shell of hydration around the protein [8, 79, 83, 86–88]. Crystal structures showing specific cation-protein binding are known [83, 84, 89]. The prevalence of protein-cation binding is not well understood, mainly because crystal structures of halophilic proteins are not able to distinguish between salt and water. To distinguish between sodium ions and water (which both have 10 electrons), data on its coordination geometry is required, which requires a structure of high resolution (below 2.4 A) [8]. 

Recently, Qvist et al. have suggested that, despite crystal structures, halophilic proteins do not have increased waters of hydration due to their greater negative charge [90]. They studied a mutant (Kx6E) of a domain in protein L (immunoglobulin G binding B1 domain) from *Streptococcus magnus*, which contained a number of salt-dependent features seen with normal halophiles (large negative charge and salt-dependent folding and stability). Using an ^17^O magnetic spin relaxation technique to monitor water associating with the protein or returning to more mobile bulk solvent, they determined that there was no difference in the amount of water bound to the halophilic over the mesophilic versions of protein L [90]. Furthermore, homology-modeled structures of halophilic dihydrofolate reductases show a similar number of hydrogen bonding networks as their nonhalophilic counterparts [86]. This raises questions on how acidic residues, then, are able to keep halophilic proteins soluble. In explaining the hydrating shell of waters seen in crystal structures, Madern et al. note that crystalizing conditions for proteins involve salting-out conditions, which cause the exclusion of salt and improve water binding [84]. The role of the acidic residues in a halophilic protein may be to increase the proteins flexibility by having a large number of nearby negative charges that repel each other [8]. The repelling charges would make it easier for a halophilic protein to change its conformation despite having a more rigid hydrophobic core (discussed below).

### 6.2. Decreased Hydrophobic Residues

Other than the larger number of acidic residues in halophilic proteins, bioinformatics studies of halophilic protein sequences have shown that they also contain different hydrophobic residues than mesophilic protein sequences. Using the known crystal structures of 15 pairs of halophilic and nonhalophilic proteins, Siglioccolo et al. determined that the hydrophobic contact in the core of halophilic proteins, exposed to molar concentrations of inorganic salt, is consistently smaller than that in mesophilic proteins (but, interestingly, not for halophilic proteins that are exposed to the organic salts) [91]. They propose that the lower hydrophobic contact in the core may counterbalance the increased strength of hydrophobic interactions in high salt concentrations [91]. Most halophilic proteins contain less of the large, aromatic hydrophobic amino acids [85]. In the homology-modeled structure of halophilic dihydrofolate reductase, there was a decrease in the number of large hydrophobic amino acids, and a reduction of the enzyme core was observed [86]. Weaker hydrophobic interactions due to smaller hydrophobic residues can increase the flexibility of protein in high salt, since it prevents the hydrophobic core from becoming too rigid [8].

### 6.3. Salt-Dependent Folding

An important advance in understanding halophilic protein adaptation has been the evidence that these proteins rely on salt to fold [92]. This research demonstrates that salt adaptation by halophiles is not only to have proteins that survive the high salt environment but that actually utilize it to function [8]. Our study of the cysteinyl-tRNA synthetase in *H. salinarum* NRC-1 shows how the enzyme not only folds from increasing salt concentrations, but it also becomes more stable and resists thermal denaturation (paper in preparation).

Salt-dependent folding may have been important for very early proteins. The typical amino acid adaptations seen in halophiles (greater acidic residues and smaller hydrophobic amino acids) have also been observed recently in constructed prebiotic proteins [93]. There are, currently, ten known amino acids that could have been created without biosynthetic pathways: alanine, aspartic acid, glutamic acid, glycine, isoleucine, leucine, proline, serine, threonine, and valine. Research by Longo et al. shows that a foldable set of these amino acids leads to a protein with halophilic features and could use high salt concentrations to fold. This suggests that a halophilic environment may have been important for biogenesis [93].

### 6.4. Halophilic Peptide Insertions

Protein adaptations to high salt are not always found throughout the entire protein sequence. In some cases, halophilicity has been significantly increased by a peptide insertion in the protein [16, 18, 94, 95]. These insertions typically contain a large number of acidic amino acids, and, as seen with cysteinyl-tRNA synthetase from *H. salinarum* NRC-1, the insertion greatly increased the catalytic turnover of the enzyme [18]. Serinyl-tRNA synthetase in *Haloarcula marismortui* also has an insertion sequence, speculated to improve enzyme flexibility [94, 96]. Ferredoxin from the same organism was shown to have an N-terminal extension that contained 15 negatively charged amino acids. This insertion is thought to improve the enzyme's solvent-accessible surface area [83, 84, 97]. These insertion sequences are proposed to have a number of possible functions and could be a way to quickly impart halophilic adaptations to a protein, evolutionarily [97].

### 6.5. Possible Industrial Applications

Halophilic proteins, so far, have found little use in industry, but there is much interest in finding an application for salt-functioning enzymes. One of these possible applications for halophilic enzymes is in treating highly saline wastewater, such as the waste created by the pickling industry, which has a saline content up to 10%. A number of other possible industrial applications for halophiles have been recently reviewed [98]. 

Some current work has gone into changing the halophilic features of some enzymes. Ishibashi et al. were able to raise and lower the salt-dependent refolding of *H. salinarum *nucleoside diphosphate kinase with only one amino acid substitution [99]. Mutating asparagine-111 to leucine (N111L) eliminated a hydrogen bond between basic dimeric units of the protein, supposedly making the formation of the functional enzyme more dependent on hydrophobic interactions. This modified the enzyme's optimum activity from 0.45 M NaCl to 1.35 M NaCl, since a higher salt concentration improves the hydrophobic interactions in the nucleoside diphosphate kinase mutant. They were also able to create the reverse effect by substituting glycine 114 to arginine (G114R). This created a new hydrogen bond between basic dimeric units and required less salt to form a functional protein [99]. Tokunaga et al. were able to impart halophilic properties to the same enzyme from the nonhalophilic *Pseudomonas aeruginosa* by only changing two adjacent residues from alanine to glutamic acid [100]. If improving an enzyme's activity in salt is as simple as changing one or two residues, or adding an insertion peptide, this means it could soon be easy to modify almost any protein to function in extreme concentrations of salt for industrial purposes.

## 7. Haloalkaliphilic Proteins

Because halophilic environments vary in pH, subsets of these environments are highly alkaline. A number of haloalkaliphilic species have been discovered in soda lakes in Egypt, Kenya, China, India, and the western United States [101]. All archaeal alkaliphiles are halophiles [102, 103]. Protein adaptations to alkaline pH in haloalkaliphiles are subtle, due to the fact that these organisms have cellular mechanisms to maintain a more neutral pH in their cytoplasm, usually within a range from 7 to 8.5 [104]. A complex cellular envelope, with a large number of glycosylated proteins, helps maintain a neutral intracellular pH [3, 105]. Also, it appears that protein adaptations to pH in haloalkaliphiles are secondary to their halophile adaptations. It was observed that the proteins from haloalkaliphiles contained a high proportion of acidic residues that is typically seen with halophilic proteins [3, 106]. Currently, there is no commercial use of archaeal haloalkaliphilic enzymes, though a number of enzymes from bacterial alkaliphiles have found use in industry, including proteases, cellulases, lipases, xylanase, pectinases, and chitinases [104]. 

## 8. Summary of Archaeal Adaptations

To illustrate these various protein adaptations, we surveyed differences, with homology modeling, among extremophiles using the enzyme cysteinyl-tRNA synthetase (CysRS). This enzyme catalyzes a highly conserved reaction, the coupling of the amino acid cysteine to its cognate tRNA, which is then used by the ribosome for protein synthesis. Because of its importance in translation, the structure of CysRS is highly conserved, and the regions of the protein sequence that are involved in tRNA binding, anticodon recognition, and catalysis are identical among all organisms. Differences in the models of CysRS between extremophiles highlight the types of adaptations that are seen in these organisms.

Homology models were made using MODELLER [107] with the *E. coli* CysRS-tRNA crystal structure (PDB: 1U0B, [10]) as a template and the amino acid sequence of CysRS from representative halophilic, thermophilic, and psychrophilic organisms. 

**Figure 1:**
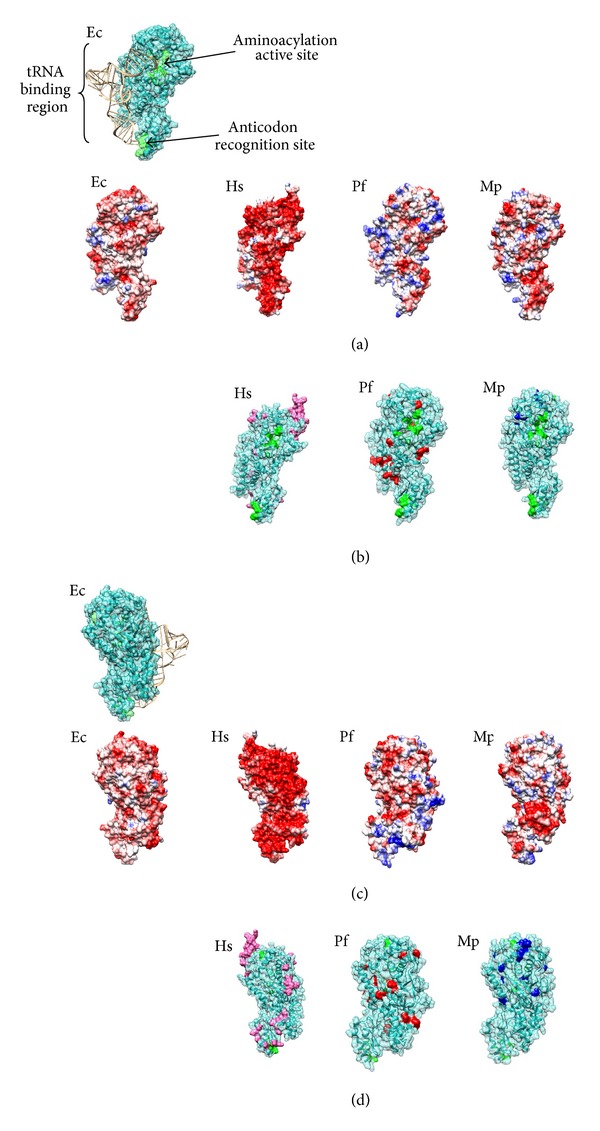
Graphical view of cysteinyl-tRNA synthetase with extremophilic protein adaptations. The homology models of *Halobacterium salinarum* (Hs), *Pyrococcus furiosus* (Pf), and *Methanolobus psychrophilus* (Mp) CysRS were generated based on the structure of *Escherichia coli* CysRS (see text for details). In the upper corner, the crystal structure of the Ec CysRS (PDB 1U0B, [10]) is provided for orientation and description of the enzyme's features. (a) and (c) Coulombic surface map of the models on the tRNA side and back of the molecule, respectively. The Coulombic surface maps the amino acid electrostatic potential (according to Coulomb's law) on surface residues: red is a negative potential, blue is a positive potential, and white indicates a relatively nonpolar potential. (b) and (d) The conserved features (in green) and unique adaptations highlighted on the surface of the models on the tRNA side and back of the molecule, respectively. The corresponding adaptations have been noted in the sequence alignment in Figure  S1 (See Figure S1 in the Supplementary Material available online at http://dx.doi.org/10.1155/2013/373275). Unique features are highlighted in different colors for the different extremes: halophilic adaptations are in pink, the thermophilic adaptations are in red, and the psychrophile adaptations are in blue. The molecular graphics were created with the USCF Chimera package [11].

The sequences used for the alignments and the models were *E. coli*, AP_001173.1, *H. salinarum* sp. NRC-1, NP_280014.1, *P. furiosus*, NP_578753.1, and *M. psychrophilus*, WP_015053952.1. MODELLER generated the following data regarding the models: *H. salinarum* was 39% identical to the *E. coli* CysRS and generated a GA341 score of 1, *P. furiosus* was 48% identical to the *E. coli* CysRS and generated a GA341 score of 1, and *M. psychrophilus* was 44% identical to the *E. coli* CysRS and generated a GA341 score of 1. A GA341 score of 1 is the highest score generated by MODELLER and indicates an acceptable model. The models were then aligned in VMD [108] to further refine the models. No energy minimization was done. Rendering was done using Chimera [11]. All models are drawn with a Coulombic surface map (Figures 1(a) and 1(c)) and a customized homology map (Figures 1(b) and 1(d)). The Coulombic surface map colors the amino acid electrostatic potential (according to Coulomb's law) on surface residues.

As can be seen in the Coulombic surface model of *E. coli* (Ec) CysRS, there is relatively even distribution of positive and negative charges, which is typical of a mesophilic, non-extremophilic protein structure. Highlighted in green on the models of the protein are the conserved regions of CysRS required for proper enzyme function.

The most dramatic change from the Ec CysRS Coulombic surface model is in the halophilic model (Hs), which displays a substantial negative potential from many acidic acid residues (aspartic acid and glutamic acid) and residues with an overall negative surface potential. This is the most common feature of halophilic proteins and enzymes. In the homology model and supplementary figure S1 of the halophilic CysRS, a peptide insertion, which is an additional 20 residues, is near the enzyme's active site. By having the insertion at this location, it is thought that it imparts additional flexibility to the enzyme around the active site [18]. In the back of the molecule, extra acidic residues dot the surface, which might function to pull positively charged ions away from the active site and tRNA binding site. 

The thermophilic CysRS model (Pf) displays a more basic and positively charged surface compared to Ec and also possesses a larger hydrophobic core seen near the active site. These features are generally associated with thermophilic proteins. The homology model and supplementary figure S1 highlights additional cysteine, proline, hydrophobic, and charged residues (in red). These residues, which are unique to the thermophilic enzyme compared to the other organisms, are seen on both sides of the enzyme, possibly indicating that these features would provide greater overall stability to the molecule. 

The psychrophilic CysRS (Mp) surface potential model shows a small reduction in surface charge, despite an unexpected acidic patch on the back of the molecule. The reduced charge is consistent with the common psychrophilic adaptation of increased surface hydrophobic residues. Other unique features observed in the homology model and supplementary figure S1 were additional glycines and hydrophobic patches (blue). A majority of these adaptations are proximal to the active site of the protein, which could impart greater flexibility in this region, improving catalytic activity at lower temperatures.

## 9. Concluding Statements

In this review we have discussed the major protein adaptations observed in archaeal organisms that thrive in vastly different extreme environments. While not all adaptations are known, it appears that, for some proteins, subtle changes in the amino acid composition are all that is needed to remain functional in an extreme environment. These differences are reflected as changes in charge, hydrophobicity, and subtle changes in structure. It is also clear that the organisms have evolved ways to manipulate these changes to optimize the protein or enzyme activity. These adaptations allow the organism and their proteins to take advantage of their environment. This has led to much interest in understanding these extreme adaptations and in manipulating these changes to find applications for these biological molecules.

## Supplementary Material

Supplementary Figure: Protein sequence alignment for the archaeal cysteinyl-tRNA synthetases. The sequences represented above are: Halobacterium. salinarum (Hs), Pyrococcus furiosis (Pf), Methanlobus psychrophilus (Mp) CysRS and Escherichia coli CysRS (Ec). These sequences have been choosen to be our "model" proteins for archaeal adaptation. This alignment was constructed from a larger alignment, which used 3 examples of each protein adaptation category and was built using HMMER3 (see supplemental text) [110]. Features that are conserved in the CysRS, like the HIGH and KMSKS domain, are highlighted in green. The features that were judged to be unique to their adaptation were highlighted in the following colors: halophilic features- pink, thermophilic features- red and psychrophilic features- blue.

## References

[1] Kletzin A (2007). *General Characteristics and Important Model Organisms*.

[2] Schleper C, Jurgens G, Jonuscheit M (2005). Genomic studies of uncultivated archaea. *Nature Reviews Microbiology*.

[3] Falb M, Pfeiffer F, Palm P (2005). Living with two extremes: conclusions from the genome sequence of *Natronomonas pharaonis*. *Genome Research*.

[4] Baker-Austin C, Dopson M (2007). Life in acid: pH homeostasis in acidophiles. *Trends in Microbiology*.

[5] Sharma A, Kawarabayasi Y, Satyanarayana T (2012). Acidophilic bacteria and archaea: acid stable biocatalysts and their potential applications. *Extremophiles*.

[6] Grant WD (2004). Life at low water activity. *Philosophical Transactions of the Royal Society B*.

[7] Ebel C, Costenaro L, Pascu M (2002). Solvent interactions of halophilic malate dehydrogenase. *Biochemistry*.

[8] Mevarech M, Frolow F, Gloss LM (2000). Halophilic enzymes: proteins with a grain of salt. *Biophysical Chemistry*.

[9] Wright DB, Banks DD, Lohman JR, Hilsenbeck JL, Gloss LM (2002). The effect of salts on the activity and stability of *Escherichia coli* and *Haloferax volcanii* dihydrofolate reductases. *Journal of Molecular Biology*.

[10] Hauenstein S, Zhang C-M, Hou Y-M, Perona JJ (2004). Shape-selective RNA recognition by cysteinyl-tRNA synthetase. *Nature Structural and Molecular Biology*.

[11] Pettersen EF, Goddard TD, Huang CC (2004). UCSF chimera—a visualization system for exploratory research and analysis. *Journal of Computational Chemistry*.

[12] Cacciapuoti G, Fuccio F, Petraccone L (2012). Role of disulfide bonds in conformational stability and folding of 5'-deoxy-5'-methylthioadenosine phosphorylase II from the hyperthermophilic archaeon *Sulfolobus solfataricus*. *Biochimica et Biophysica Acta*.

[13] Szilágyi A, Závodszky P (2000). Structural differences between mesophilic, moderately thermophilic and extremely thermophilic protein subunits: results of a comprehensive survey. *Structure*.

[14] Vieille C, Zeikus GJ (2001). Hyperthermophilic enzymes: sources, uses, and molecular mechanisms for thermostability. *Microbiology and Molecular Biology Reviews*.

[15] Connaris H, Chaudhuri JB, Danson MJ (1999). Expression, reactivation, and purification of enzymes from *Haloferax volcanii* in *Escherichia coli*. *Biotechnology and Bioengineering*.

[16] Evilia C, Ming X, Dassarma S, Hou Y-M (2003). Aminoacylation of an unusual tRNACys from an extreme halophile. *RNA*.

[17] Yonezawa Y, Tokunaga H, Ishibashi M, Taura S, Tokunaga M (2003). Cloning, expression, and efficient purification in *Escherichia coli* of a halophilic nucleoside diphosphate kinase from the moderate halophile *Halomonas* sp. #593. *Protein Expression and Purification*.

[18] Evilia C, Hou Y-M (2006). Acquisition of an insertion peptide for efficient aminoacylation by a halophile tRNA synthetase. *Biochemistry*.

[19] Bae E, Phillips GN (2004). Structures and analysis of highly homologous psychrophilic, mesophilic, and thermophilic adenylate kinases. *The Journal of Biological Chemistry*.

[20] Kumar S, Nussinov R (2004). Different roles of electrostatics in heat and in cold: adaptation by citrate synthase. *ChemBioChem*.

[21] Tehei M, Zaccai G (2007). Adaptation to high temperatures through macromolecular dynamics by neutron scattering. *FEBS Journal*.

[22] Powers ET, Balch WE (2013). Diversity in the origins of proteostasis networks—a driver for protein function in evolution. *Nature Reviews Molecular Cell Biology*.

[23] Dill KA, Ozkan SB, Shell MS, Weikl TR (2008). The protein folding problem. *Annual Review of Biophysics*.

[24] Fitzkee NC, Fleming PJ, Gong H, Panasik N, Street TO, Rose GD (2005). Are proteins made from a limited parts list?. *Trends in Biochemical Sciences*.

[25] Tomazic SJ, Klibanov AM (1988). Mechanisms of irreversible thermal inactivation of *Bacillusα*-amylases. *The Journal of Biological Chemistry*.

[26] Mayer F, Küper U, Meyer C (2012). AMP-forming acetyl coenzyme a synthetase in the outermost membrane of the hyperthermophilic crenarchaeon *Ignicoccus hospitalis*. *Journal of Bacteriology*.

[27] Bräsen C, Urbanke C, Schönheit P (2005). A novel octameric AMP-forming acetyl-CoA synthetase from the hyperthermophilic crenarchaeon *Pyrobaculum aerophilum*. *FEBS Letters*.

[28] Ingram-Smith C, Smith KS (2007). AMP-forming acetyl-CoA synthetases in Archaea show unexpected diversity in substrate utilization. *Archaea*.

[29] Del Vecchio P, Elias M, Merone L (2009). Structural determinants of the high thermal stability of SsoPox from the hyperthermophilic archaeon *Sulfolobus solfataricus*. *Extremophiles*.

[30] Park JT, Song HN, Jung TY (2013). A novel domain arrangement in a monomeric cyclodextrin-hydrolyzing enzyme from the hyperthermophile *Pyrococcus furiosus*. *Biochimica Et Biophysica Acta*.

[31] Park K-H, Kim T-J, Cheong T-K, Kim J-W, Oh B-H, Svensson B (2000). Structure, specificity and function of cyclomaltodextrinase, a multispecific enzyme of the *α*-amylase family. *Biochimica et Biophysica Acta*.

[32] Vihinen M (1987). Relationship of protein flexibility to thermostability. *Protein Engineering*.

[33] Cacciapuoti G, Porcelli M, Bertoldo C, De Rosa M, Zappia V (1994). Purification and characterization of extremely thermophilic and thermostable 5’-methylthioadenosine phosphorylase from the archaeon *Sulfolobus solfataricus*. Purine nucleoside phosphorylase activity and evidence for intersubunit disulfide bonds. *The Journal of Biological Chemistry*.

[34] Boutz DR, Cascio D, Whitelegge J, Perry LJ, Yeates TO (2007). Discovery of a thermophilic protein complex stabilized by topologically interlinked chains. *Journal of Molecular Biology*.

[35] Woycechowsky KJ, Raines RT (2003). The CXC motif: a functional mimic of protein disulfide isomerase. *Biochemistry*.

[36] Wilkinson B, Gilbert HF (2004). Protein disulfide isomerase. *Biochimica et Biophysica Acta*.

[37] Karshikoff A, Ladenstein R (2001). Ion pairs and the thermotolerance of proteins from hyperthermophiles: a “traffic rule” for hot roads. *Trends in Biochemical Sciences*.

[38] Hendsch ZS, Tidor B (1994). Do salt bridges stabilize proteins? A continuum electrostatic analysis. *Protein Science*.

[39] Chan C-H, Yu T-H, Wong K-B (2011). Stabilizing salt-bridge enhances protein thermostability by reducing the heat capacity change of unfolding. *PLoS ONE*.

[40] Fukuchi S, Nishikawa K (2001). Protein surface amino acid compositions distinctively differ between thermophilic and mesophilic bacteria. *Journal of Molecular Biology*.

[41] Lee C-F, Makhatadze GI, Wong K-B (2005). Effects of charge-to-alanine substitutions on the stability of ribosomal protein L30e from *Thermococcus celer*. *Biochemistry*.

[42] Liu YF, Zhang N, Liu X Molecular mechanism underlying the interaction of typical Sac10b family proteins with DNA. *PLoS ONE*.

[43] Mamat B, Roth A, Grimm C (2002). Crystal structures and enzymatic properties of three formyltransferases from archaea: environmental adaptation and evolutionary relationship. *Protein Science*.

[44] Breitung J, Borner G, Scholz S, Linder D, Stetter KO, Thauer RK (1992). Salt dependence, kinetic properties and catalytic mechanism of *N*-formylmethanofuran:tetrahydromethanopterin formyltransferase from the extreme thermophile *Methanopyrus kandleri*. *European Journal of Biochemistry*.

[45] Unsworth LD, Van Der Oost J, Koutsopoulos S (2007). Hyperthermophilic enzymes—stability, activity and implementation strategies for high temperature applications. *FEBS Journal*.

[46] De Champdoré M, Staiano M, Rossi M, D’Auria S (2007). Proteins from extremophiles as stable tools for advanced biotechnological applications of high social interest. *Journal of the Royal Society Interface*.

[47] Fang J, Zhang L, Bazylinski DA (2010). Deep-sea piezosphere and piezophiles: geomicrobiology and biogeochemistry. *Trends in Microbiology*.

[48] Hay S, Evans RM, Levy C (2009). Are the catalytic properties of enzymes from piezophilic organisms pressure adapted?. *ChemBioChem*.

[49] Boonyaratanakornkit BB, Park CB, Clark DS (2002). Pressure effects on intra- and intermolecular interactions within proteins. *Biochimica et Biophysica Acta*.

[50] Di Giulio M (2005). A comparison of proteins from *Pyrococcus furiosus* and *Pyrococcus abyssi*: barophily in the physicochemical properties of amino acids and in the genetic code. *Gene*.

[51] Mombelli E, Shehi E, Fusi P, Tortora P (2002). Exploring hyperthermophilic proteins under pressure: theoretical aspects and experimental findings. *Biochimica et Biophysica Acta*.

[52] Consonni R, Santomo L, Fusi P, Tortora P, Zetta L (1999). A single-point mutation in the extreme heat- and pressure- resistant Sso7d protein from *Sulfolobus solfataricus* leads to a major rearrangement of the hydrophobic core. *Biochemistry*.

[53] Fusi P, Goossens K, Consonni R (1997). Extreme heat- and pressure-resistant 7-kDa protein P2 from the archaeon *Sulfolobus solfataricus* is dramatically destabilized by a single-point amino acid substitution. *Proteins*.

[54] Sun MMC, Caillot R, Mak G, Robb FT, Clark DS (2001). Mechanism of pressure-induced thermostabilization of proteins: studies of glutamate dehydrogenases from the hyperthermophile *Thermococcus litoralis*. *Protein Science*.

[55] Rosenbaum E, Gabel F, Durá MA (2012). Effects of hydrostatic pressure on the quaternary structure and enzymatic activity of a large peptidase complex from *Pyrococcus horikoshii*. *Archives of Biochemistry and Biophysics*.

[56] Simonato F, Campanaro S, Lauro FM (2006). Piezophilic adaptation: a genomic point of view. *Journal of Biotechnology*.

[57] Abe F, Horikoshi K (2001). The biotechnological potential of piezophiles. *Trends in Biotechnology*.

[58] Huang Y, Krauss G, Cottaz S, Driguez H, Lipps G (2005). A highly acid-stable and thermostable endo-*β*-glucanase from the thermoacidophilic archaeon *Sulfolobus solfataricus*. *Biochemical Journal*.

[59] Golyshina OV, Timmis KN (2005). Ferroplasma and relatives, recently discovered cell wall-lacking archaea making a living in extremely acid, heavy metal-rich environments. *Environmental Microbiology*.

[60] Jackson BR, Noble C, Lavesa-Curto M, Bond PL, Bowater RP (2007). Characterization of an ATP-dependent DNA ligase from the acidophilic archaeon “*Ferroplasma acidarmanus*” Fer1. *Extremophiles*.

[61] Magnet S, Blanchard JS (2004). Mechanistic and kinetic study of the ATP-dependent DNA ligase of *Neisseria meningitidis*. *Biochemistry*.

[62] Rohwerder T, Gehrke T, Kinzler K, Sand W (2003). Bioleaching review part A: progress in bioleaching: fundamentals and mechanisms of bacterial metal sulfide oxidation. *Applied Microbiology and Biotechnology*.

[63] Luisa Tutino M, Di Prisco G, Marino G, De Pascale D (2009). Cold-adapted esterases and lipases: from fundamentals to application. *Protein and Peptide Letters*.

[64] Smalas AO, Leiros HK, Os V (2000). Cold adapted enzymes. *Biotechnology Annual Review*.

[65] Dong X, Chen Z (2012). Psychrotolerant methanogenic archaea: diversity and cold adaptation mechanisms. *Science China Life Sciences*.

[66] Feller G (2010). Protein stability and enzyme activity at extreme biological temperatures. *Journal of Physics*.

[67] Cavicchioli R, Thomas T, Curmi PMG (2000). Cold stress response in Archaea. *Extremophiles*.

[68] Dassarma S, Capes MD, Karan R (2013). Amino acid substitutions in cold-adapted proteins from *Halorubrum lacusprofundi*, an extremely halophilic microbe from antarctica. *PLoS ONE*.

[69] Karan R, Capes MD, DasSarma P (2013). Cloning, overexpression, purification, and characterization of a polyextremophilic *β*-galactosidase from the Antarctic haloarchaeon *Halorubrum lacusprofundi*. *BMC Biotechnology*.

[70] Thomas T, Cavicchioli R (1998). Archaeal cold-adapted proteins: structural and evolutionary analysis of the elongation factor 2 proteins from psychrophilic, mesophilic and thermophilic methanogens. *FEBS Letters*.

[71] Saunders NFW, Thomas T, Curmi PMG (2003). Mechanisms of thermal adaptatation revealed from genomes of the anatarctic *Archaea Methanogenium frigidum* and *Methanacoccoides burtonii*. *Genome Research*.

[72] D’Amico S, Gerday C, Feller G (2001). Structural determinants of cold adaptation and stability in a large protein. *The Journal of Biological Chemistry*.

[73] Georlette D, Damien B, Blaise V (2003). Structural and functional adaptations to extreme temperatures in psychrophilic, mesophilic, and thermophilic DNA ligases. *The Journal of Biological Chemistry*.

[74] Giaquinto L, Curmi PMG, Siddiqui KS (2007). Structure and function of cold shock proteins in archaea. *Journal of Bacteriology*.

[75] Russell RJM, Gerike U, Danson MJ, Hough DW, Taylor GL (1998). Structural adaptations of the cold-active citrate synthase from an Antarctic bacterium. *Structure*.

[76] Aghajari N, Van Petegem F, Villeret V (2003). Crystal structures of a psychrophilic metalloprotease reveal new insights into catalysis by cold-adapted proteases. *Proteins*.

[77] D’Amico S, Sohier JS, Feller G (2006). Kinetics and energetics of ligand binding determined by microcalorimetry: insights into active site mobility in a psychrophilic *α*-amylase. *Journal of Molecular Biology*.

[78] Hasan F, Shah AA, Hameed A (2006). Industrial applications of microbial lipases. *Enzyme and Microbial Technology*.

[79] Karan R, Capes MD, Dassarma S (2012). Function and biotechnology of extremophilic enzymes in low water activity. *Aquatic Biosystems*.

[80] Zhang G, Huihua G, Yi L (2013). Stability of halophilic proteins: from dipeptide attributes to discrimination classifier. *International Journal of Biological Macromolecules*.

[81] Britton KL, Baker PJ, Fisher M (2006). Analysis of protein solvent interactions in glucose dehydrogenase from the extreme halophile *Haloferax mediterranei*. *Proceedings of the National Academy of Sciences of the United States of America*.

[82] Dym O, Mevarech M, Sussman JL (1995). Structural features that stabilize halophilic malate dehydrogenase from an archaebacterium. *Science*.

[83] Frolow F, Harel M, Sussman JL, Mevarech M, Shoham M (1996). Insights into protein adaptation to a saturated salt environment from the crystal structure of a halophilic 2Fe-2S ferredoxin. *Nature Structural Biology*.

[84] Madern D, Ebel C, Zaccai G (2000). Halophilic adaptation of enzymes. *Extremophiles*.

[85] Zhang G, Ge H (2013). Protein hypersaline adaptation: insight from amino acids with machine learning algorithms. *The Protein Journal*.

[86] Kastritis PL, Papandreou NC, Hamodrakas SJ (2007). Haloadaptation: insights from comparative modeling studies of halophilic archaeal DHFRs. *International Journal of Biological Macromolecules*.

[87] Soppa J (2006). From genomes to function: haloarchaea as model organisms. *Microbiology*.

[88] Tadeo X, López-Méndez B, Trigueros T, Laín A, Castaño D, Millet O (2009). Structural basis for the aminoacid composition of proteins from halophilic archea. *PLoS Biology*.

[89] Richard SB, Madern D, Garcin E, Zaccai G (2000). Halophilic adaptation: novel solvent protein interactions observed in the 2.9 and 2.6 Å resolution structures of the wild type and a mutant of malate dehydrogenase from *Haloarcula marismortui*. *Biochemistry*.

[90] Qvist J, Ortega G, Tadeo X, Millet O, Halle B (2012). Hydration dynamics of a halophilic protein in folded and unfolded states. *Journal of Physical Chemistry B*.

[91] Siglioccolo A, Paiardini A, Piscitelli M, Pascarella S (2011). Structural adaptation of extreme halophilic proteins through decrease of conserved hydrophobic contact surface. *BMC Structural Biology*.

[92] Müller-Santos M, de Souza EM, Pedrosa FDO (2009). First evidence for the salt-dependent folding and activity of an esterase from the halophilic archaea *Haloarcula marismortui*. *Biochimica et Biophysica Acta*.

[93] Longo LM, Lee J, Blaber M (2013). Simplified protein design biased for prebiotic amino acids yields a foldable, halophilic protein. *Proceedings of the National Academy of Sciences of the United States of America*.

[94] Taupin CM-J, Härtlein M, Leberman R (1997). Seryl-tRNA synthetase from the extreme halophile *Haloarcula marismortui*. Isolation, characterization and sequencing of the gene and its expression in *Escherichia coli*. *European Journal of Biochemistry*.

[95] Taupin CM-J, Leberman R (1999). Archaeabacterial seryl-tRNA synthetases: adaptation to extreme environments and evolutionary analysis. *Journal of Molecular Evolution*.

[96] Zaccai G, Cendrin F, Haik Y, Borochov N, Eisenberg H (1989). Stabilization of halophilic malate dehydrogenase. *Journal of Molecular Biology*.

[97] Marg B-L, Schweimer K, Sticht H, Oesterhelt D (2005). A two-*α*-helix extra domain mediates the halophilic character of a plant-type ferredoxin from Halophilic Archaea. *Biochemistry*.

[98] Oren A (2010). Industrial and environmental applications of halophilic microorganisms. *Environmental Technology*.

[99] Ishibashi M, Hayashi T, Yoshida C (2013). Increase of salt dependence of halophilic nucleoside diphosphate kinase caused by a single amino acid substitution. *Extremophiles*.

[100] Tokunaga H, Arakawa T, Tokunaga M (2008). Engineering of halophilic enzymes: two acidic amino acid residues at the carboxy-terminal region confer halophilic characteristics to *Halomonas* and *Pseudomonas* nucleoside diphosphate kinases. *Protein Science*.

[101] Enache M, Itoh T, Fukushima T, Usami R, Dumitru L, Kamekura M (2007). Phylogenetic relationships within the family *Halobacteriaceae* inferred from rpoB′ gene and protein sequences. *International Journal of Systematic and Evolutionary Microbiology*.

[102] Ollivier B, Caumette P, Garcia J-L, Mah RA (1994). Anaerobic bacteria from hypersaline environments. *Microbiological Reviews*.

[103] Grant WD (2004). *Half A Lifetime in soda lakes*.

[104] Horikoshi K (1999). Alkaliphiles: some applications of their products for biotechnology. *Microbiology and Molecular Biology Reviews*.

[105] Siddaramappa S, Challacombe JF, De Castro RE (2012). A comparative genomics perspective on the geneticcontent of the alkaliphilic haloarchaeon Natrialbamagadii ATCC 43099T. *BMC Genomics*.

[106] Giménez MI, Studdert CA, Sánchez JJ, De Castro RE (2000). Extracellular protease of *Natrialba magadii*: purification and biochemical characterization. *Extremophiles*.

[107] Eswar N, Webb B, Marti-Renom MA (2006). Comparative protein structure modeling using Modeller. *Current Protocols in Bioinformatics*.

[108] Humphrey W, Dalke A, Schulten K (1996). VMD: visual molecular dynamics. *Journal of Molecular Graphics*.

[109] Goecks J, Nekrutenko A, Taylor J, Galaxy Team T (2010). Galaxy: a comprehensive approach for supporting accessible, reproducible, and transparent computational research in the life sciences. *Genome Biology*.

[110] Eddy SR (1998). Profile hidden Markov models. *Bioinformatics*.

