# Supplementary material for: Protein Adaptations in Archaeal Extremophiles
Source: Archaea. 2013 Sep 16;2013:373275. doi: 10.1155/2013/373275 (PMC3787623; doi:10.1155/2013/373275)
Supplement: Supplementary file 1 — Supplementary Figure: Protein sequence alignment for the archaeal cysteinyl-tRNA synthetases. The sequences represented above are: Halobacterium. salinarum (Hs), Pyrococcus furiosis (Pf), Methanlobus psychrophilus (Mp) CysRS and Escherichia coli CysRS (Ec). These sequences have been choosen to be our "model" proteins for archaeal adaptation. This alignment was constructed from a larger alignment, which used 3 examples of each protein adaptation category and was built using HMMER3 (see supplemental text) [110]. Features that are conserved in the CysRS, like the HIGH and KMSKS domain, are highlighted in green. The features that were judged to be unique to their adaptation were highlighted in the following colors: halophilic features- pink, thermophilic features- red and psychrophilic features- blue. [file 373275.f1.pdf]

Supplementary Information for Reed et al., "Protein Adaptations in Archaeal Extremophiles"

Figure S1 is meant to provide protein sequence information on the similarity and conserved features of these cysteinyl-tRNA synthetases. The alignment in figure S1 was created using the HMMER3 algorithm [99]. This was done using a Hidden Markov model, containing 3 of each organism (3 Bacteria, 3 Archaeal Halophiles, 3 Archaeal thermophiles and 3 Psychrophiles) to align the four sequences in figure S1. The additional sequences used, along with the four used in the homology model (see above), were *Methanobacterium* sp. Maddingley MBC34 (WP_008514564.1), *Acidianus hospitalis* (WP_013775295.1), *Picrophilus torridus* (WP_011176885.1), *Archaeoglobus fulgidus* (WP_0101877918.1), *Ignicoccus hospitalis* (WP_012123134.1), *Ferroplasma acidarmanus* (WP_009887938.1), *Haloarcula marimostui* (YP_135935.1), *Haloferax volcanii* (YP_003535180.1), *Bacillus cereus* (NP_976417.1), *Lactococcus lactis* (YP_007509177.1), *Planococcus antarcticus* (WP_006828899.1), and *Pseudoaltermonas haloplanktis* (WP_011328723.1). Features of extremophilic adaptations were highlighted on the alignment.

```
Ec  1    ------MLKIFNTLTRQKEEFKPIHAG-EVGMYVCGITVYDLCHIGHGRTFVAFDVVARYLRFLGYKLKYVRNITDIDDK 73
Mp  1    ----MA-LRVYNTLTREMEEFVPLHGK-KVNMYVCGPTVYDHCHLGHARSYISFDVMRRYLSYRGYDVRYISNVTDVDDK 74
Pf  1    MPRGKNMLKVYNTLTKQKEEFKPLREG-EVKMYVCGPTVYDYPHLGHARTYIAFDVIRRYLEHKGYTVLMVMNFTDIDDK 79
Hs  1    ---MTQ--YVSNTRSGEQEAFEPDDPE-NVLVYTCGLTVSDDAHLGHARLWVQSDVMTRWLSHAGYGVRHVQNVTDVNEK 74

Ec  74   IIKRANEN--GESFVAMVDRMIAEMHKDFDALNILRPDMEPRATHHIAEIIELTEQLIAKGHAYVADNGDVMFDVPTD-P 150
Mp  75   IINRAKET--GEDPFDLSARFTRSFIEDMTSLRVKKPDVQPTVTGHITEIVDAIKLLIIREFAYPTPEGNVYYDLRRSKE 152
Pf  80   IIKRARET--GEDPKELAERFIKIFLEDMEALKVKPADIYPRVTDHIDDIIEFIGKLKEKGYAYEG-SDGIYFEVKKF-P 155
Hs  75   IVARVGADGLGDTEAAVAAHYTQSVIDDMRALNLARADVYPRVSEHVPEIIDLIGDLVDAGYAYEA-GGSVYFDVRRF-E 152

Ec  151  TYGVLSRQDLDQLQAG--ARVDV---VDDKRNPMDFVLWKMSKEGE-------------------P----SWPSPWGAGR 202
Mp  153  KIGIISHQTEEGLMEGSGARIDV---EKDKRYPLDFVLWKSSSEGQ-------------------P----GWDSPWSRGR 206
Pf  156  EYGKLSGVKIEDLQKG--ARVEP---GEGKKNPEDFALWKKAKPGE-------------------P----KWDSPWGEGR 207
Hs  153  EYGALSGQQVDELDPQ--GPDAE---QAEKRHPADFALWKAGGVSPDDANTHRDDELPPLDGERGQ----TWASPWGEGR 223

Ec  203  PGWHIECSAMNCKQLGNHFDIHGGGSDLMFPHHENEIAQSTCA-HDGQYVNYWMHSGMVMVDREKMSKSLGNFFTVRDVL 281
Mp  207  PGWHIECSAMSMKYGSQQLDIHGGGADLIFPHHEAEIHQSEGCTGKHPFSKYWMHNGFLTIDKEKMSKSLGNFFTIKQVL 286
Pf  208  PGWHIECSVMSSKYLGESFDIHGGGNDLIFPHHENEIAQSEAC-FGHEWVKYWLHTGFVMVKGEKMSKSLGNFVTIRELL 286
Hs  224  PGWHIECSAMAMTHLDDHIDIHVGGQDLVFPHHENEIAQSEAA-SGERFADHWLHVRLLETDGEKMSSSLGNFFTVSNAV 302

Ec  282  KYYDAETVRYFLMSGHYRSQLNYSEENLKQARAALERLYTALRGTDKTVAP---------------AGGEAFEARFIEAM 346
Mp  287  EEFPPEVIRFFILNTHYRNTIDFSKMHLQEAGRAYERIANTIVNVRYAIEN----APEEDNDSGLSDEISEARDQFTISM 362
Pf  287  KRYEPEVIRFFVLQKHYRSPLEYTEEGLQHAKNNLQRLYNTLENIRVALRNAEISYTWGELEFKTYEIIREGKRKFYEAM 366
Hs  303  AERGPNVVRMLLVSTSYTQRQTYSEATVSEATQRWERLQRAHERAADAIDSV--AAHAKPADDALRTAVADARGEFAAAM 380

Ec  347  DDDFNTPEAYSVLFDMAREVNRLKAEDMAAA----NAMASHLRKL-SAVLGLLEQEPE--AFLQSGAQADDSEVAEIEAL 419
Mp  363  DEDFNTREALANLFIFSRKVNAIISGSSPGR-PALEAILDFFSEI-DEVLGVFGKDIS-GVT---EGTSRGLSDKDIDAM 436
Pf  367  DDDFNTAEALKAVFEVANAINKYLTEANKPKESILRKALEFFKIV-SEVFGVFEDYFREET------KEREESEKLIELL 439
Hs  381  RADFNTRAAVSALLELASAVNRHVDGTDTYDYQGLHDAVDAFETLGGDVLGLQFDDGA-GE------DAVSLADDVIDLV 453

Ec  420  IQQRLDARKAKDWAAADAARDRLNEMGIVLEDGPQGTTWRRK--- 461
Mp  437  IQQREEARRSKDWQRSDAIRDELKEKGIVIEDGKEGVRWRRV--- 478
Pf  440  VEVRKQLRKEKRYELADMIREELKKLGIQLEDRGSETTWKRIIT- 483
Hs  454  LDVREQERTAGNYERADDLRDRLEALGVSVEDTDDGATVRR---- 494
```

Figure S1. Protein sequence alignment for the archaeal cysteinyl-tRNA synthetases. The sequences represented above are: *Halobacterium. salinarum* (Hs), *Pyrococcus furiosis* (Pf), *Methanlobus psychrophilus* (Mp) CysRS and *Escherichia coli* CysRS (Ec). These sequences have been choosen to be our "model" proteins for archaeal adaptation. This alignment was constructed from a larger alignment, which used 3 examples of each protein adaptation category and was built using HMMER3 (see supplemental text) [110]. Features that are conserved in the CysRS, like the HIGH and KMSKS domain, are highlighted in green. The features that were judged to be unique to their adaptation were highlighted in the following colors: halophilic features- pink, thermophilic features- red and psychrophilic features- blue.
